# Organizational readiness to implement a care model in primary care for frail older adults living at home in Sweden

**DOI:** 10.3389/frhs.2022.958659

**Published:** 2022-11-24

**Authors:** Kristin Thomas, Petra Dannapfel

**Affiliations:** ^1^Division of Society and Health, Department of Health, Medicine and Caring Sciences, Faculty of Medicine and Health, Linköping University, Linköping, Sweden; ^2^Region Östergötland, Linköping, Sweden

**Keywords:** implementation science, organizational readiness, primary health care, qualitative methods, care pathways

## Abstract

**Background:**

The demographic change of an aging population constitutes a challenge for primary care organizations worldwide. The systematic implementation of preventative and proactive care models is needed to cope with increased care demands.

**Objective:**

To investigate the organizational readiness in primary care to implement a new care model to prevent hospitalization among frail older adults.

**Method:**

Individual qualitative interviews with health care staff investigated organizational readiness at seven primary care units in Sweden. A semi-structured interview guide was used during the interviews and included broad questions on individual and collective readiness to change. Directed content analysis and organizational readiness to change theory were used in data analysis.

**Results:**

Positive beliefs among staff such as perceived benefits and compatibility with existing values contributed to a strong commitment to implement the new care model. However, perceptions such as unclear task demands, limited resources and concerns about new collaborative structures challenged implementation.

**Conclusions:**

The findings emphasize implementation as an inter-organizational phenomenon, especially for holistic practices that span across multiple health care providers and disciplines. Furthermore, implementing care models in healthcare may require a change of culture as much as a change of practice.

## Introduction

The world's population is aging and the number of people over the age of 65 years is estimated to more than double by the year 2050 (1). As the population ages, the number of frail older adults will increase with changed health care demands as a result, for example, care of multi-morbidity, chronic diseases as well as acute conditions (2, 3). Primary health care has been considered an ideal setting to address the needs of the frail older adults at risk of hospitalization (4). However, the health and care needs are not always identified in a timely manner because the health care system has a predominantly reactive orientation, i.e., acting when disease, injury or symptoms have occurred.

The importance of achieving a more standardized, individualized and proactive health care for frail older adults in Sweden provided the impetus for implementing a new primary care model called “Focused Primary Care” (FPC) during 2017-2019 (5, 6). The model includes a tool that identifies older adults that are at-risk of hospitalization and prompts primary care to systematically appraise medical status and health and social care needs among this population. The FPC model strives to be proactive (by identifying and reaching out to at-risk frail older adults); holistic (addressing social, psychiatric, functional, and medical concerns) and individualized (interactive care plans are created by a multi-professional team, patients, and family). There is an increased demand for shared responsibility and collaboration to meet overall needs. Older adults are over-represented with regarding both length of stay and frequency of hospital admissions (7). In addition, care has been found to be fragmented and poorly coordinated which may have further challenged the implementation of preventative care for this group (8).

Within implementation theory, several factors are typically proposed to determine implementation outcomes such as the characteristics of an innovation (e.g., the complexity of the care model); the individuals that implement the innovation (e.g., attitudes toward the care model among staff); contextual factors (e.g., financial resources) and the strategies that are employed (e.g., training offered to staff) (9). Furthermore, these determinants for implementation act on multiple levels of an organization, interact and together contribute to implementation success or failure. So-called organizational readiness to change has been recognized and shown to be a central aspect for successful implementation. Indeed, theory and empirical studies indicate that the readiness for change, to be central for subsequent implementation processes and outcomes (10–14) in terms of both individual staff members and collective group levels. The Organizational readiness to change theory conceptualize readiness to change as the shared experience of the ability and willingness to change in an organization. The theory further posit that this shared sense of readiness is determined by the extent to which individuals feel committed to change, confident in the collective ability to change, value the change as important and worthwhile as well as the extent to which they perceive that there are sufficient resources for change in the organization (11).

### Aim

To investigate the organizational readiness to implement a new care model for frail older adults in nine primary care units in Sweden.

## Methods

### Study design

A qualitative interview study including interviews with physicians and nurses from nine primary care units. Directed content analysis (15) and the Organizational readiness to change theory was used in data collection and analysis (11).

### Theoretical framework

Organizational readiness to change (ORC) has been argued to be a critical success factor for the implementation of new innovations (12). The ORC theory was developed by Weiner (11) and used to inform interview questions and data analysis. The theory conceptualizes implementation of change as collective, coordinated efforts carried out by organizational members. Thus, “organizational readiness” to implement change is the shared psychological state in which organizational members feel committed to change and confident in their collective ability to change. Determinants of organization readiness to change consist of change valence (how much organization members value the specific change and why) and situational assessment (task knowledge, resource availability and situational factors). These two determinants affect change commitment and change efficacy (the collective cognitive appraisal of the situational factors) taken together this predict the organizational readiness to change.

### Setting

In Sweden, individual primary care units are responsible for offering preventative, primary and secondary care to the population living in their specific geographical area and registered patients. Primary care is also responsible for care in the home for older adults for example medical treatment and rehabilitation. Although primary care is not responsible for social services, the implementation of the new care model (Focused primary care) prompted collaboration with various actors outside of primary care for instance social services. Nine primary care units were invited and took part in interviews. All nine units were expected to within the FPC trial (5) implement a new care model in routine primary care. All nine units were located in Region Östergötland in the south of Sweden.

### Participants

Purposive sampling was employed, inclusion criterion was health care professionals who were expected to adopt the new care model and had a critical role in the implementation process. Eligible individuals were identified in collaboration with the manager at each unit. The ambition was to recruit both physicians and nurses as these two professions were to have different roles in the implementation of the care model. Eligible individuals were invited through an e-mail that described the aim of interviews and information relating to participating e.g., confidentiality. A total of 18 individuals (nine physicians and nine registered nurses) were identified as eligible and invited to take part in interviews. Out of these, 12 individuals accepted to take part, and were interviewed (five physicians and seven registered nurses; one man and 11 women).

### Data collection

Data collection was conducted at an early phase of implementation to capture key aspects of perceived readiness to change at the units. A semi-structured interview guide was used that aimed to capture (1) how the care model was perceived and understood. e.g., how new ways of working would affect current routines, (2) individual readiness to change, e.g., motivation and skills; and (3) organizational readiness to change, e.g., resources to implement the care model at the workplace. The interview guide was first piloted in an interview to determine its ability to capture data relevant to the study aims. The questions were perceived to be informative and valid, thus no major revisions were made, and the test interview was included in the analysis. Data collection was done by authors KT and PD (both PhD, female and postdoc researchers at the time with experience in qualitative methods) and two members of the larger FPC project group (both research assistants, female with experience in qualitative methods). Interviews were conducted in the workplace of each participant and lasted between 40 and 60 min. At the end of each interview, the interviewer asked if there was anything that had not been elucidated. All interviews were recorded and transcribed verbatim by a professional transcriber. Informed consent was obtained before each interview after the participants had been given written information about the study and informed that participation was confidential and voluntary, and that they could withdraw at any time during or after the interview. No compensation was given to participants and no relationship was established between interviewer and participants prior study commencement.

### Data analysis

The interview data were analyzed using directed content analysis in accordance with Hsieh and Shannon (15). Content analysis is a method for analyzing texts based on empirical data that are explorative and descriptive. The use of a deductive category application and use of existing theory can help to focus the research question to predict variables of interest and the relationships between variables (15). Directed content analysis is a structured process for coding data using an existing theory or previous research (15). In this study, ORC theory (11) was used to analyze data.

As a first step, both authors read all transcripts repeatedly to gain an understanding of the whole dataset. The transcripts were then coded by the authors separately, which entailed coding and categorizing the data according to each construct of the ORC theory, both in terms of determining initial coding and relationship between the codes. In the next stage, the authors discussed the interpretation of the data in relation to ORC theory and compared their coding. The discussions continued until no inconsistencies existed and a shared understanding was reached to prevent researcher bias and strengthen the internal validity (16). Data that were deemed not to correspond to the OTC theory in the coding phase were coded later on in the analysis process and if relevant, labelled inductivally (15). Representative quotations were identified to report the findings throughout the analysis. As a last step, quotations were translated from Swedish to English by the authors.

## Results

The data showed that the new care model was perceived by nurses and physicians to include four standardized steps: identification of at-risk individuals, care planning, execution- and follow-up of care (Table 1). The data further indicated that the primary care units were at different stages of the implementation process at the time of the interviews. Some units had started to prepare for implementing the care model which was illustrated by for example allocating staff roles and responsibilities, whereas other units were at an earlier phase, perhaps only started to reflect on making organizational changes. However, all respondents were able to reflect and talk about the implementation of the care model at their unit.

**Table 1:** A step-by-step description of the care model according to data.

**Step**	**Actions**	**Actors**
**Identification**		
Prediction	Identification of at-risk older adults based on algorithms/matching criteria using data from the regional care register	Project management team
Compilation	A list of at-risk adults is compiled for each participating primary care unit	Project management team
Distribution	A list of at-risk adults is distributed to the manager at each primary care unit	Managers (primary care units)
Review	Mapping of physicians responsible for each frail older adult who has been identified as at-risk Mapping of current care situation	Registered nurses (PC^†^)
Prioritization	Prioritizing how patients should be contacted	Registered nurses (PC^†^) and physician
**Care planning**		
Patient interview	The frail older adult is contacted via telephone where standardized questions is used to explore physical, mental and social health, general care needs and current medications	Registered nurses (PC^†^) and patients
Examination	Visit to the primary care unit for an examination. If the patient is not able to attend, home visits are carried out	Registered nurses (PC^†^) and patients
Assessment rounds	Generation of interactive care plans	Registered nurses (PC^†^ and M^‡^ as needed) Physicians
**Execution of care**		
Treatment/action	Care is carried out according to the interactive care plans	Registered nurses (PC^†^ and M^‡^) Physicians Rehabilitation professions
**Follow-up of care**		
Follow-up of treatment and actions	Health care professionals talk with patients and follow up care needs and outcomes	Registered nurses (PC^†^)
Revisions of care plans		Physicians Patients

^†^PC, primary health care;^‡^M, municipality (care in the home).

The informants described that a single nurse or a small group of nurses were put in charge of implementing procedures. Designated nurses had the responsibility to book appointments with patients, coordinating the work on generating interactive care plans and monitoring patients over time. At the primary care units, mainly registered nurses and physicians actively worked with the care model. However, fully executing care plans also required liaising with stakeholders outside the primary care unit, e.g., municipality nurses (home care) and physiotherapists. Below follows results for each construct of the Organizational Readiness for Change theory (11): change valence, change commitment, situational assessment and change efficacy.

### Change valance

Change valence refers to the extent organizational members perceived it necessary to implement the care model, expressed it to be important, beneficial, and worthwhile (11). The data showed that nurses and physicians were positive toward implementing the care model. Positive regard was expressed as beliefs that the new model will standardize the care process, increase work satisfaction among staff, and be beneficial for the patients. Furthermore, informants expressed the belief that the new care model would be highly relevant for frail older adults and that the new way of working would enable them to better meet the care expectations of this patient group.

“*I have huge expectations, I felt that this way of working was needed when I worked in the municipality. The elderly is a large group. I love to work with elderly; it is the best thing I know … They don't need to explain their life story all over again; this way of working has huge advantages for the elderly*” Respondent 2, registered nurse.

Furthermore, informants expressed that the model enabled opportunities for patients to have a more central role in the healthcare process in terms of communicating their needs and preferences during care planning. The informants also expressed that they believed that patients would benefit from having an increasingly centered role in their care plan. Respondents expressed that the new care model required them to adapt a new approach whereby patient preferences were considered and addressed in a more systematic way compared to before.

“*You have a more structured way of thinking and what to pay attention to. Previously, it was more fragmentary and not put into a wider context. The patients' response has not been the driving force, but rather what is written in the journal and my own and the nurses' experience. This method is more centered and based on the patients' response to the questions... it's different from how we worked before. Then there was more focus on medical assessment and what to do more or less in relation to that*” Respondent 5, physician.

The informants also expressed beliefs that implementing the care model could promote health care staff to employ a more holistic approach which was thought to be beneficial for patients. One example was working with the interactive care plans which required health care staff to consider multiple aspects of patients' situation including medical needs as well as home and social life. To fully employ a holistic approach to care thus required closer collaboration with, predominantly physicians, but also other health professions such as physiotherapists, occupational therapists or home care nurses.

“*It is another type of collaboration. Before, you had specific questions (to the physician) but now we sit down together and discuss what I and the patient have talked about … It is rewarding; you feel that you are doing a good job … The patients are satisfied when they leave*” Respondent 6, registered nurse.

Furthermore, working holistically was expressed by nurses and physicians as something worthwhile and important. To understand the health and care needs from a holistic perspective was thought to not only benefit patients but also improve general work satisfaction among staff because these aspects were in accordance with core values and professional role expectations.

“*You don't have so much time to work proactively. One thought I had was, will I generate more jobs when we already have trouble finding physicians. But if we work proactively, we might prevent people from getting sick and we will decrease the inflow in the long run; that is a good way to work*” Respondent 4, registered nurse.

Lastly, change valence was represented by the belief that the new care model would improve the care process for the patient group as a whole. The new care model was believed to enable continuity of care, including allowing patients to have one point of contact at their primary care unit. Respondents emphasized that a major strength of the new care model was providing primary care with structured way of working with frail older adults.

“*It will be easier for the patient. They know that the nurse will call on Mondays and they can talk. It increases the feeling of safety and continuity. It also means you don't need to call an extra time. You can get to know your patient and that is extra important for this patient group*” Respondent 2, registered nurse.

### Change commitment

Change commitment referred to organizational members' willingness and motivation to pursue the course of action involved in implementing the change (11). The level of change commitment is determined by the degree of change valence. Several primary care units had already started to plan for and improve their work with frail elderly persons. Thus, for them, the care model as a new way of working was legitimate and the purpose well sanctioned.

“*We had made our own prediction list and then this research study came along. We lost some time because we already had our own plan and had to re-think; but we all wanted to do this*” Respondent 5, physician.

Change commitment was expressed among the respondents by the continuous effort to invest in implementing the new care model despite, e.g., limited resources or resistance among colleagues. Also, respondents described engaging in relational work with colleagues and patients to push for implementation or avoid resistance.

“*Absolutely, we are positive and see this assignment as our responsibility to a very high degree*” Respondent 1, physician.

“*You need to have time; we have that, we schedule this. It is me and one more nurse here who have half a day to work with this … It increases the pressure elsewhere, but that was there before this change too. It is always stressful, but we agreed to be involved and we want to prioritize this*” Respondent 6, registered nurse.

However, the data showed that negative experiences of previous implementation efforts or practice change to some extent compromised change commitment. For example, one nurse mentioned that she was tired of constantly being involved in new initiatives especially since several initiatives over the years had not been successfully implemented.

### Situational assessment

Situational assessment is proposed to determine change efficacy and consists of task knowledge, perceived resource availability, and other situational factors (11). That is, did the informants know what it would take to implement the new care model and did they believe the care model could be successfully implemented at their unit given their current resources for change. The data showed that resources to implement change was perceived as both sufficient (experience in implementing change) and insufficient (staff resources). Specifically, resources to implement change were characterized in the data by the availability of time and staff and competency to implement change. Although respondents expressed a belief that there was sufficient competency and experience to implement change, they also voiced concern regarding the limited resources that were available in terms of staff and time.

“*You find a concept that seems good and investigate how they have solved it somewhere else, with regard to finding resources, and find they put in a lot of resources. Then you are supposed to do the same thing but with no resources … that permeates what is expected from primary care*” Respondent 9, physician.

Regarding how nurses and physicians understood what was expected from them (task knowledge and demands), the respondents expressed the care model was somewhat unclear, for instance regarding roles and responsibilities. Sound knowledge about the model and what each step meant regarding workload and responsibility for staff was highlighted as important for successful implementation. Also, informants described how the timing of communicating roles and responsibilities were important, and clear from start.

“*Knowledge about the model is required. If it this is not communicated properly, you don't know what is expected from you … Before you have the prediction list, it is not a good idea to talk too much about the model because that will create more anxiety than clarity. … If I were to ask if they [colleagues] know what is expected of them in the different steps today, 80-90% would say that they don't have a clear picture. It has to be totally clear if it is to work successfully in practice*” Respondent 1, physician.

Implementing the new care model entailed collaborating with new partners in a more structured way, including patients, family, and the municipality. The informants foresaw challenges with these new collaborative structures because of limited previous experience as well as limited resources and a rigid organizational structure. Collaboration beyond colleagues at the primary care unit was expressed as a central component of the care model to fully employ a holistic approach to care and to be able to monitor care outcomes over time. Respondents appraised how the new care model affected them and their work situation.

“*I will work with five different physicians; that means it is going to be quite fragmentized and difficult. It feels like a more general organizational change might be needed for this to work well, but we'll see; collaboration I believe will be important*” Respondent 3, physician.

“*We should collaborate more with the municipality and work differently but we don't have the resources … you could work full time with only this and still feel that you lack time. There are many elderly who are ill*” Respondent 10, registered nurse.

### Change efficacy

Change efficacy refers to organizational members' cognitive appraisal of three determinants of implementation capability: task demands, resource availability, and situational factors (11). The data showed that the situational assessment in terms of poor understanding of the new way of working and perceptions of limited resources could limit change efficacy. Also, nurses and physicians expressed that collaborating with professions outside of the primary care unit or the municipality could be challenging. Although respondents expressed motivation and commitment to change, aspects that challenge their change efficacy were present:

“*Many of these patients have home care, which makes things a little tricky; how do we do it? We can ask home care to do the interviews from the lists, but they have no obligation to do so; the interface between us is tricky*” Respondent 9, physician.

Poor understanding of the new care model among staff negatively influenced change efficacy. For example, one informant expressed concern regarding their limited experience working with the interactive care plans. Respondents also highlighted that their primary care unit was already under pressure even before embarking on implementing the new care model. Informants described limited organizational capacity.

“*Today the situation is already strained in primary care so that is a bit frustrating*” Respondent, 1, registered nurse.

## Discussion

This qualitative study explored organizational readiness to implement a new care model for frail older adults in primary care in Sweden, using the Organizational readiness to change theory (11). Overall, the findings showed a strong commitment to change among nurses and physicians, which was characterized by positive beliefs and expectations regarding the care model. However, findings also suggested that unclear task demands, perceptions of limited resources, and concerns about new collaborative structures compromised change efficacy.

Shared commitment to make a practice change and implement the new care model stemmed from beliefs that the model would bring change that was needed and worthwhile. Indeed, the findings showed that both nurses and physicians could see valued benefits of the care model on both patient and staff levels, and that the new way of working resonated core values: an increasingly proactive and holistic approach to care. Care for frail older adults requires assessments of physical, psychological, and social capacity and function. That is, it is essential to adopt a proactive, multi-professional, and holistic approach rather than focusing on the treatment of disease. Adopting a holistic care approach, including maintaining acceptable levels of functioning and not just preventing death and disease, could have facilitated the respondents' commitment to the change and has been mentioned as a significant cornerstone of health interventions for an aging population (17). The findings showed that the staff members believed that the new care model could offer a valuable framework to work in this way. A review on the effectiveness of care pathways and models showed that they can indeed facilitate standardized care, documentation, professional behavior change, and decision making (18). In addition, a mixed-method study investigating the implementation of a similar care model in Netherlands showed that a similar model provided a useful and feasible structure to deliver geriatric primary care and contributed to similar positive aspects to those found in this study, such as, work satisfaction among staff and patients feeling increasingly acknowledged by clinicians and that patients' care needs were met in a more adequate way (2).

Indeed, the approach of the new care model echoed core values among organizational members which could be an important facilitator for implementation. For example, Self-determination theory posits that the higher an individual values a specific change, and the more intrinsic the motivation is, the more likely it is that change (implementation) will occur (19). Previous research supports the idea that commitment based on “want to” rather than “need to” or “have to” represents a higher level of commitment. It has been shown that individuals with intrinsic commitment also display more cooperative behavior (e.g., volunteering for tasks) and championing behavior (e.g., promoting implementation) (20). Most of the respondents expressed that they “wanted to” use the intervention and exhibited cooperative behavior and took explicit responsibility for the implementation. Also, informants described that they promoted the value of the change to colleagues, thus performing a championing behavior. Early implementation research showed that change champions indeed influence implementation outcomes (21, 22). Recent qualitative research looking at important champion characteristics in successful implementation efforts proposed that long-term commitment, willingness to promote the innovation, credibility, capacity, and social capital were all qualities that could facilitate and drive implementation forward (23). Thus, the model's ability to tap into core values and subsequent champion behaviors among physicians and nurses would probably facilitate implementation in a significant way.

However, findings on beliefs in the collective capability to implement the model revealed some challenges. Informants perceived task demands to be unclear, resources to be limited, and expressed concerns about the need for new collaborative structures that reached outside of the care unit. For example, informants expressed a limited understanding of the roles and responsibilities of the whole chain of care (from identification of at-risk patients to follow-up of care). A scoping review (24) on conditions for implementing care coordination highlight the central theme of complexity, in terms of both care complexity (multiple care providers) and case complexity (patients with multimorbidity). The authors highlight the potential need to both reduce complexity and embrace it to achieve good and equal care. Similarly, our findings suggest that case complexity was mainly embraced illustrated by for instance commitment to a more holistic approach to care. On the other hand, the complexity of care and the need for new collaborative structures was not embraced to the same extent. Indeed, our findings showed concerns that the new care model required collaboration across primary-, home- and social care which was an infrastructure that was perceived to not yet be in place. Strickland (25) draws on system theory and emphasizes that organizational change may not be separated from other organizations but are rather closely connected to their environment. The value of a system perspective on organizational readiness to change is apparent in this type of innovations because it is greatly affected by structures, processes and culture of multiple organizations and divisions. That is, the primary care organization cannot in isolation implement the full range of care because they only have control of one part of the care model (in this case identification and assessment). To fully understand organizational readiness to implement care models such as the one in our study therefore needed a more comprehensive system-approach to readiness.

Thus, the findings suggested discrepancies between the strong commitment of the staff on the one hand and perceived lack of resources and capabilities of the primary care system and culture on the other hand. ORC theory posits that change commitment (willingness) and change efficacy (assessed ability) are inter-related and can influence each other (11). For example, beliefs in poor abilities to implement a change could impair strong commitment for change or vice versa. Scaccia and colleagues (12) propose that organizational readiness for change encompass motivation to implement an innovation, general capacity for change as well as innovation-specific capacity for change. Our findings suggest strong motivation to implement the care model but however, that innovation-specific capacity in terms of change in culture was limited. Indeed, to implement a new approach to care, shifting from predominantly reactive care to a proactive approach may need a cultural change within the primary care system. For example, the care model was described as a long-term ambition which could potentially clash with the short-term realities of everyday practice. More importantly, it has been proposed that high readiness in one area (e.g., motivation) will not compensate for poor readiness in another area (e.g., innovation-specific capacity) (12) which confirms that discrepancies in readiness seen in our findings would need to be carefully considered to optimize conditions for implementation.

Organizational members perceived that they had limited resources to implement new practice routines, and lack of time was mentioned as a hindrance to implementation. However, several respondents mentioned that the intervention was sanctioned and supported by managers, and most participants had time set aside even when the unit was under pressure. Time restraints is an often-cited barrier for implementation (3, 26). Schein (27) highlighted however the importance of leaders being consistent in imposing what is valued, even during times of organizational strain, arguing that communicating priorities can be more important than added resources.

Lastly, the findings support the notion that organizational readiness to change is a multi-level and multi-faceted construct. Determinants of readiness was found at individual, group and unit levels for example perceptions of the new way of working, multi-professional collaboration and management support. Furthermore, additional determinants were identified for instance difficulties in collaborating with stakeholders outside of the immediate care unit. Thus, the findings showed that this “collective” extends to inter-organizational relationships, patients, and family.

### Methodological considerations

To employ a theoretical framework in analysis was useful to understand and structure the data in terms of identifying key aspects of readiness for change for example beliefs among health care professionals that the new care model was needed (change valence in ORC). The research design included multiple primary care units implementing the same new care model. This design allowed for multiple examples of contexts and processes to study and compare, which we believe strengthened the data. However, a limitation was that only physicians and nurses from the primary care units were included. Considering the holistic approach of the care model, it could have been valuable to also investigate the perspectives of other professions such as social workers or psychologists or other actors for instance patients or municipality nurses. However, we adopted a primary care unit approach whereby the organizational readiness of the units was investigated rather than the implementation process of the care model in general. Furthermore, the study was conducted within the Swedish health care system which may have limited the transferablity of the findings to health care organizations outside of Sweden. Finally, the limited number of interviews that were feasible to conduct can have reduced the credibility of the data. However, individuals eligible for interview were restricted. Since only nine units were expected to implement the care model, this resulted in 18 individuals eligible for interview.

## Conclusions

Implementing a new care model for frail older adults requires collaborative efforts from primary care, municipalities, patients, and their family members. This study emphasizes the importance of considering implementation as an inter-organizational phenomenon, especially for interventions that span across different health care providers. Readiness to change must thus be considered across the whole patient journey, which requires a deeper understanding of inter-organizational processes. This study further indicates that implementing a proactive, holistic, and multi-professional approach to care demands a change of culture in primary care as much as a change of practice. Further, that implementation required new skills, i.e., working in multi-professional teams, and change of mindset toward a proactive population management and understanding change management. The evolution of patient care models will also need resources; primary care cannot alone front the cost of the transformation needed to support this new approach to care if implementation is to be successful.

## Data availability statement

The raw data supporting the conclusions of this article will be made available by the authors upon reasonable request.

## Ethics statement

This qualitative study and data collection was part of a larger study which were reviewed and approved by Regional Ethical Review Board at Linköping University (2016/347-31). Participants gave informed consent prior interviews.

## Author contributions

KT contributed to the planning of the study, conducted interviews, analyzed the data, and wrote the first draft of the manuscript. PD contributed to the planning of the study, conducted interviews, and analyzed the data. Both authors read and approved the last version of the manuscript.

## Funding

The study was financed by grants from Strategic Research Area Healthcare Welfare, Östergötland County Council, and Linköping University (e.g., 2016-347-31, main applicant KT).

## Conflict of interest

The authors declare that the research was conducted in the absence of any commercial or financial relationships that could be construed as a potential conflict of interest.

## Publisher's note

All claims expressed in this article are solely those of the authors and do not necessarily represent those of their affiliated organizations, or those of the publisher, the editors and the reviewers. Any product that may be evaluated in this article, or claim that may be made by its manufacturer, is not guaranteed or endorsed by the publisher.

## References

[1] World Health Organization. World Health Statistics. (2022). Available online at: https://www.who.int/data/gho/publications/world-health-statistics (accessed May 31, 2022).

[2] Metzelthin SF Daniëls R van Rossum E Cox K Habets H de Witte LP. Nurse-led interdisciplinary primary care approach to prevent disability among community-dwelling frail older people: a large-scale process evaluation. Int J Nurs Stud. (2013) 50:1184–96. 10.1016/j.ijnurstu.2012.12.016 23384696

[3] Stijnen MMN Jansen MWJ Duimel-Peeters IGP Vrijhoef HJM. Nurse-led home visitation program to improve health-related quality of life and reduce disability among potentially frail community-dwelling older people in general practice: a theory-based process evaluation. BMC Fam Pract. (2014) 15:173. 10.1186/s12875-014-0173-x 25344322 PMC4213477

[4] Muntinga ME Van Leeuwen KM Schellevis FG Nijpels G Jansen A. From concept to content: Assessing the implementation fidelity of a chronic care model for frail, older people who live at home. BMC Health Serv Res. (2015) 15:18. 10.1186/s12913-014-0662-6 25608876 PMC4312437

[5] Marcusson J Nord M Johansson M.M, Alwin J, Levin LÅ, Dannapfel P, et al. Proactive healthcare for frail elderly persons: Study protocol for a prospective controlled primary care intervention in Sweden. BMJ Open. (2019) 9:1–8. 10.1136/bmjopen-2018-027847 31122995 PMC6538001

[6] Nord M., Östgren CJ, Marcusson J, Johansson M. Staff experiences of a new tool for comprehensive geriatric assessment in primary care (PASTEL): A focus group study: Primary care staff experiences of geriatric assessment. Scand J Prim Health Care. (2020) 38 132–145. 10.1080/02813432.2020.1755786 32349567 PMC8570711

[7] Condelius A Edberg AK Jakobsson U Hallberg IR. Hospital admissions among people 65+ related to multimorbidity, municipal and outpatient care. Arch Gerontol Geriatr. (2008) 46 41–55. 10.1016/j.archger.2007.02.005 17403548

[8] Larsen T Falk H Bångsbo A. Onödig slutenvård av sköra äldre. En kunskapsöversikt (Unnecessary hospitalization of frail elderly: a systematic review). Götegorgsregionen FoU väst (2013). Available online at: https://goteborgsregionen.se/download/18.2c9665f617704cb69f3278a0/1614004244882/On%C3%B6dig%20slutenv%C3%A5rd%20av%20sk%C3%B6ra%20%C3%A4ldre.pdf (accessed November 9, 2022).

[9] Nilsen P. Making sense of implementation theories, models and frameworks. Implement Sci. (2015) 10 1–13. 10.1186/s13012-015-0242-0 25895742 PMC4406164

[10] Matthew-Maich N Harris L Ploeg J Markle-Reid M Valaitis R Ibrahim S Gafni A Isaacs S. Designing, implementing, and evaluating mobile health technologies for managing chronic conditions in older adults: a scoping review. JMIR MHealth UHealth. (2016) 4 e29. 10.2196/mhealth.5127 27282195 PMC4919548

[11] Weiner BJ A. theory of organizational readiness for change. Implement Sci. (2009) 4:67. 10.1186/1748-5908-4-67 19840381 PMC2770024

[12] Scaccia JP Cook BS Lamont A Wandersman A Castellow Katz J. A practical implementation science heuristic for organizational readiness: R = MC2. J. Community Pychology. (2015) 43:484–501. 10.1002/jcop.21698 26668443 PMC4676714

[13] Hagedorn HJ Heideman PW. The relationship between baseline organizational readiness to change assessment subscale scores and implementation of hepatitis prevention services in substance use disorders treatment clinics: a case study. Implement Sci. (2010) 5:46. 10.1186/1748-5908-5-46 20546584 PMC2902416

[14] Kelly P Hegarty J Barry J Dyer KR Horgan A. A systematic review of the relationship between staff perceptions of organizational readiness to change and the process of innovation adoption in substance misuse treatment programs, J. Subst Abuse Treat. (2017) 80:6–25. 10.1016/j.jsat.2017.06.001 28755775

[15] Hsieh HF Shannon SE. Three approaches to qualitative content analysis. Qual Health Res. (2005) 15:1277–88. 10.1177/1049732305276687 16204405

[16] Patton MQ. Qualitative Research & Evaluation Methods: Integrating Theory and Practice. 4th. Sage publications (2014).

[17] Suzman Beard JR Boerma T Chatterji S. Health in an ageing world—what do we know? Lancet. (2015) 385:484–6. 10.1016/S0140-6736(14)61597-X 25468156

[18] Allen D Gillen E Rixson L. Systematic review of the effectiveness of integrated care pathways: What works, for whom, in which circumstances? Int J Evid Based Healthc. (2009) 7:61–74. 10.1111/j.1744-1609.2009.00127.x 21631848

[19] Ryan RM Deci EL. Self-determination theory and the facilitation of intrinsic motivation, social development, and well-being. Am Psychol. (2000) 55:68–78. 10.1037/0003-066X.55.1.68 11392867

[20] Herscovitch L Meyer JP Commitment Commitment to Organizational Change : Extension of a Three-Component Model. J Appl Psychol. (2002)87:474–87. 10.1037/0021-9010.87.3.474 12090605

[21] Goldman J Meuser J Lawrie L Rogers J Reeves S. Interprofessional primary care protocols: a strategy to promote an evidence-based approach to teamwork and the delivery of care. J Interprof Care. (2010) 24:653–65. 10.3109/13561820903550697 20919959

[22] Jaén C Crabtree B Palmer R Ferrer R Nutting P Miller W. Methods for evaluating practice change toward a patient-centered medical home. Ann Fam Med Fam Med. (2010) 8:9–20. 10.1370/afm.1108 20530398 PMC2885721

[23] Bunce AE Gruß I Davis JV Cowburn S Cohen D Oakley J. Lessons learned about the effective operationalization of champions as an implementation strategy: Results from a qualitative process evaluation of a pragmatic trial, Implement. Sci. (2020) 15:1–12. 10.1186/s13012-020-01048-1 32998750 PMC7528604

[24] Doessing A Burau V. Care coordination of multimorbidity: a scoping study. J Comorbidity. (2015) 5:15–28. 10.15256/joc.2015.5.39 29090157 PMC5636034

[25] Strickland F. The Dynamics of Change: Insights into Organizational Transition from the Natural World. London: Routledge. (1998).

[26] Bleijenberg N Dam VH Drubbel I Numans ME De Wi NJ Schuurmans MJ. Development of a Proactive Care Program (U-CARE) to preserve physical functioning of frail older people in primary care. J Nurs Scholarsh. (2013) 45:230–37. 10.1111/jnu.12023 23530956

[27] Schein EH. Organizational culture. Am Psychol. (1990) 45:109–19. 10.1037/0003-066X.45.2.109

